# A Dutch cost-effectiveness analysis of fremanezumab versus best supportive care in patients with chronic migraine and inadequate response to prior preventive therapy

**DOI:** 10.1186/s12883-024-03697-x

**Published:** 2024-06-24

**Authors:** Sharon Wolters, Johannes A. Carpay, Marja H. Pronk, Karin W.M. Zuurbier, Maurice T. Driessen, Leonidas Lyras, Maarten J. Postma

**Affiliations:** 1 Department of Health Sciences, University of Groningen, University Medical Center Groningen, Hanzeplein 1, Groningen, 9713 GZ The Netherlands; 2 Asc Academics B.V., Hereweg 120, Groningen, 9725 AK The Netherlands; 3 The Migraine Clinic, Mariotteplein 60, Amsterdam, 1098 PA The Netherlands; 4 MH Pronk Health Care Consultancy Foundation, Leidsestraatweg, Woerden, 41D, 3443 BP The Netherlands; 5 Teva Netherlands B.V., Swensweg 5, Haarlem, 2031 GA The Netherlands; 6 Teva Pharmaceuticals, Piet Heinkade 107, Amsterdam, 1019 BR The Netherlands; 7 Department of Economics, Econometrics and Finance, University of Groningen, Antonius Deusinglaan 1, Groningen, 9713 AV The Netherlands

**Keywords:** Chronic migraine, Burden of disease, Calcitonin gene-related peptide, Cost effectiveness, Health technology assessment, Economic modeling, Fremanezumab, Netherlands

## Abstract

**Background:**

Chronic migraine (CM) is the most severe and burdensome subtype of migraine. Fremanezumab is a monoclonal antibody that targets the calcitonin gene-related peptide pathway as a migraine preventive therapy. This study aimed to conduct a cost-effectiveness analysis of fremanezumab from a societal perspective in the Netherlands, using a Markov cohort simulation model.

**Methods:**

The base-case cost-effectiveness analysis adhered to the Netherlands Authority guidelines. Fremanezumab was compared with best supportive care (BSC; acute migraine treatment only) in patients with CM and an inadequate response to topiramate or valproate and onabotulinumtoxinA (Dutch patient group [DPG]). A supportive analysis was conducted in the broader group of CM patients with prior inadequate response to 2–4 different classes of migraine preventive treatments. One-way sensitivity, probabilistic sensitivity, and scenario analyses were conducted.

**Results:**

Over a lifetime horizon, fremanezumab is cost saving compared with BSC in the DPG (saving of €2514 per patient) and led to an increase of 1.45 quality-adjusted life-years (QALYs). In the broader supportive analysis, fremanezumab was cost effective compared with BSC, with an incremental cost-effectiveness ratio of €2547/QALY gained. Fremanezumab remained cost effective in all sensitivity and scenario analyses.

**Conclusion:**

In comparison to BSC, fremanezumab is cost saving in the DPG and cost effective in the broader population.

**Supplementary Information:**

The online version contains supplementary material available at 10.1186/s12883-024-03697-x.

## Background

Migraine is a complex neurological disease characterized by moderate to severe headache attacks that can last many hours to days; additionally, symptoms and extreme tiredness can persist for some time following an attack [1]. Chronic migraine (CM) is the most burdensome classification and is defined by International Headache Society *International Classification of Headache Disorders, Third Edition* criteria as headache occurring on ≥ 15 days per month for > 3 months, which exhibits migraine characteristics on ≥ 8 days per month [1]. CM accounts for approximately 10–20% of migraine cases [2, 3] and has a substantial negative impact on health-related quality of life (HRQoL) [3].

Migraine is a highly prevalent condition, with an estimated 283,800 patients known to general practitioners in the Netherlands and an overall prevalence of 18.1% [4, 5]. Migraine is more commonly experienced by women (78.7% of known Netherlands patients with migraine are women), and its prevalence peaks during childbearing/working years [4, 5]. Given its prevalence and potential severity, migraine has a very high global social burden. In the Global Burden of Disease 2019 study, headache disorders were the 14th leading cause of disability-adjusted life-years [6], and in the 15–49 years age group, migraine was the top cause of years lived with disability [7]. This leads to substantial economic burden for migraine, which, in the Netherlands, is estimated at a €2.3–4.2 billion cost to society [8], but is potentially as high as €5 billion per year [9]. Much of this cost is associated with productivity losses [9], but direct healthcare costs from migraine are also large, especially for patients with CM. It was estimated in 2010 that direct medical costs for patients with CM in individual EU countries ranged from approximately €1500 in Germany to almost €4000 in the UK per patient per year [10]. Effective prevention of migraine therefore has a huge cost-saving potential for healthcare systems.

Pharmacological treatment for migraine consists of two main approaches; acute treatment to relieve the effects of migraine attacks, and preventive treatment to reduce the frequency, severity, and duration of attacks over time. Migraine prophylaxis is recommended in European guidelines for when migraine attacks cause disability on ≥ 2 days per month despite optimized acute therapy [11, 12]. More recent guidelines from the Dutch Association for Neurology note that migraine preventive pharmaceuticals, including topiramate, valproate, and onabotulinumtoxinA for CM, have generally been repurposed from other therapeutic areas [13]. These guidelines state that onabotulinumtoxinA should generally be reserved for CM patients with failure of previous migraine treatments [13].

Calcitonin gene-related peptide (CGRP) pathway treatments are a class of migraine preventive therapies targeted at the underlying pathophysiology of migraine. Fremanezumab, a humanized monoclonal antibody that selectively targets CGRP, demonstrated effective preventive treatment in patients with migraine with an inadequate response to 2–4 classes of prior preventive treatments in the Phase 3b FOCUS clinical trial [14]. Recent European Headache Federation guidelines recommend the use of CGRP pathway monoclonal antibody (mAb) migraine preventive treatments as a first-line treatment option [15].

The aim of this study was to conduct a cost-effectiveness analysis of fremanezumab from a societal perspective in the Netherlands. The primary patient population considered was patients with CM who have failed on topiramate or valproate and onabotulinumtoxinA, in line with the Netherlands reimbursed indication where fremanezumab is considered a last-line treatment (consideration of potential reimbursement criteria for episodic migraine [EM] is ongoing) [16]. Results of a post hoc subgroup analysis of the FOCUS trial in this group were recently published [17].

## Methods

### Model outline/structure and perspective

**Fig. 1:**
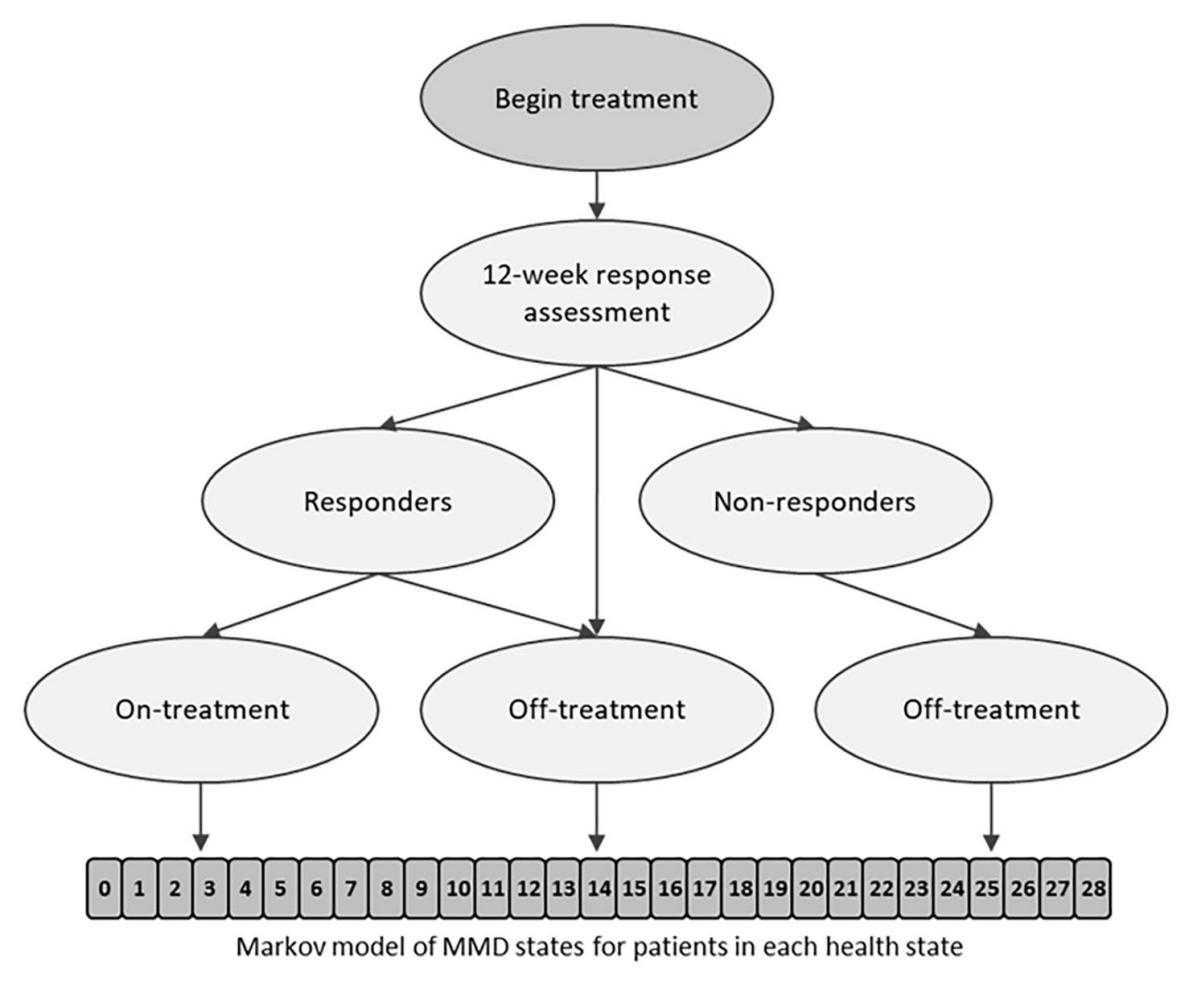
Model structure. A responder was a patient with a ≥ 30% reduction in MMD from baseline during the 12 weeks after therapy initiation. MMD, monthly migraine days

A Markov cohort simulation model was developed and used to conduct cost-effectiveness analyses of fremanezumab versus best supportive care (BSC) from a Netherlands societal perspective. The model consisted of an initial decision tree to assess response after 12 weeks of treatment (Fig. 1). At this point, patients were split based on treatment response, with responder patients continuing treatment and other patients discontinuing treatment (negative stopping rule). Patients then entered the semi-Markov part of the model, where there were two health states (on treatment; off treatment) plus an absorbing state of death (due to natural causes). Within the two main health states, patients were distributed across 29 monthly migraine days (MMD). This structure was similar to that described in other recently published models in migraine [18].

The model investigated fremanezumab as the intervention of interest, with BSC (modeled as acute migraine treatment only) chosen as the appropriate comparator for this patient population. The licensed dose of fremanezumab is either 225 mg per month or 675 mg every three months [19]; as both dosing options are equivalent in total dose, cost, efficacy, and safety profile; they were modeled in a single analysis. The model base case adhered to the Dutch recommendations for cost-effectiveness analyses (as defined by Zorginstituut Nederland [ZIN]) [20] and, in the base case, considered a societal perspective (including direct and indirect costs).

Ethics approval was not required as data were derived from previously conducted studies, for which ethical approval had been obtained [14]. The FOCUS clinical trial was used as the primary source of model efficacy inputs (monthly dosing for CM patients included a loading dose of 675 mg); previously published data and additional post hoc analyses were utilized [14]. No formal health economic analysis plan was prepared in advance. Reporting standards for this manuscript conform to the guidelines promulgated in the 2022 Consolidated Health Economic Evaluation Reporting Standards (CHEERS) [21].

### Patient population

The primary patient population consisted of patients with CM (≥ 15 monthly headache days, of which ≥ 8 were MMD) who had a documented inadequate response (defined as in the FOCUS trial: no clinically meaningful improvement after ≥ 3 months at a stable dose, discontinuation because of intolerable adverse events, or treatment contraindicated or unsuitable) [14] to topiramate or valproate and onabotulinumtoxinA within the past 10 years (referred to as the Dutch patient group [DPG]) [16]. This patient population was based on a subgroup analysis of the FOCUS data [14]. To address uncertainties from the reduced group size in this subgroup analysis, an additional supportive analysis was conducted in the full CM population of the FOCUS trial (patients with CM who had a documented inadequate response to 2–4 classes of migraine preventive therapy within the past 10 years [beta-blockers, anticonvulsants, tricyclic antidepressants, calcium channel blockers, angiotensin II receptor antagonists, onabotulinumtoxinA, and valproic acid]). Patient baseline characteristics were derived from all relevant patients in the FOCUS trial, with a mean age of 47 years and 84.1% female population at model start (82.3% female for full FOCUS CM population) [14, 17].

### Migraine day modeling and efficacy inputs

There were three distinct stages to the modeling of MMD and changes in MMD, which are described below. In brief, longitudinal curves first were fit using data from the placebo arms of FOCUS so as to generate mean MMD for the BSC arm. Next, a treatment effect for fremanezumab was then applied to the placebo data to generate mean MMD for fremanezumab. Finally, the dispersion of patients across MMD states was modeled by applying the relevant beta binomial distribution to derived mean MMD values.

To model mean MMD in the BSC arm, the placebo arm of FOCUS was used to produce curves for MMD change over time that then were applied to the BSC model arm. This analysis was conducted using an exponential function with model parameters estimated via least-squares. The exponential function was chosen as it afforded the best fit to the data versus linear or alternative nonlinear functional forms.

For the fremanezumab arm, a treatment effect was applied to BSC MMD values using mean change in MMD versus placebo from relevant analyses of FOCUS data. Migraine day reductions observed at 12 weeks were modeled to be maintained for the remainder of the assumed time horizon (lifetime in the model base case), as currently available data show no reduction in efficacy over up to 15 months of treatment with fremanezumab [22].

Several statistical modeling techniques were investigated for their ability to describe the observed patient distributions from FOCUS (analyzed separately for responders and non-responders). Specifically, longitudinal beta binomial and negative binomial models were investigated for their ability to accurately model these data (Tables S1 and S2). Model selection was based on the Bayesian Information Criterion (BIC), with the model with the lowest BIC preferred. This analysis was primarily concerned with determining which distribution provided a better fit to the observed data and the estimate of the dispersion parameter for that distribution. Based on the goodness of fit for the modeled distributions, beta binomial distributions were selected and subsequently used to estimate the dispersion of patients across migraine day states throughout the trial treatment period. Beta binomial distributions were produced separately for responder and non-responder patients; for CM and EM patients; and for treated (fremanezumab) and placebo patients (used for BSC) (Figure S1).

The final efficacy input was response rate, the proportion of patients with a ≥ 30% reduction in MMD from baseline in the 12 weeks after therapy initiation, calculated from FOCUS data for both BSC and fremanezumab [14, 17].

### HRQoL

Utilities were applied in the model based on MMD, with each MMD state associated with a utility value that was assumed to not vary over time. Migraine-Specific Quality of Life Questionnaire (MSQ) data from the FOCUS trial were considered to be the most appropriate HRQoL data to use and were mapped to the EQ-5D-3L scale to provide utility values for this model [14]. This mapping was conducted using the published algorithm of Gillard et al. [23]. The MSQ was considered better able to capture the full impact of migraine as it has a 4-week recall period, allowing the full burden across and between migraine attacks to be captured, whereas EQ-5D is an ‘on-the-day’ assessment that can miss the full impact of migraine attacks (FOCUS patients completed the EQ-5D in clinic and would be unlikely to travel if suffering an attack) [14].

The mapped EQ-5D-3L data were split into on treatment and off treatment, where off treatment consisted of all baseline data and on treatment consisted of data for patients receiving fremanezumab at both available time points from the FOCUS trial (Week 4 and Week 12). These data were fitted to a beta regression model, with model selection determined by the BIC. Parameters of the selected model were used to calculate utilities for each MMD state (Table S3).

### Costs and resource use

The model used Dutch costs in euros at 2020 prices, thereby reflecting the Dutch submission (Table 1) [24–28]. It was assumed therapy initiation was performed during a neurologist visit and further administrations were self-injections at home following nurse training. Healthcare resource use data were sourced from reports of the National Health and Wellness Survey, which included data from France, Germany, Italy, Spain, and the United Kingdom [29, 30]. Work productivity losses were estimated from published data on absenteeism and presenteeism for each MMD state [31]. The recommended friction cost method was considered [20], although the friction period does not apply to migraine as absence will be randomly divided across time and not a continuous long-term absence.

**Table 1:** Model Cost Inputs

Item	Description and derivation	2020 costs	
Resource use – fremanezumab			
Fremanezumab acquisition cost	Fremanezumab acquisition costs of €509.96 per injection converted to cost per 4-week cycle	€470.73	
Total fremanezumab costs in first cycle (initiation costs)	Costs of fremanezumab acquisition + one neurologist visit + travel costs + nurse training visit	€595.97	
Fremanezumab administration costs	Costs of self-injection at home	€0	
Fremanezumab costs per cycle	Costs of fremanezumab acquisition + administration costs	€470.73	
Resource use – medical costs			
GP visit	Costs per GP visit [24]	€39.39	
Monitoring costs	Costs of 6-monthly monitoring are applied starting from Month 9 and consist of a neurologist visit; these are then applied as costs per cycle [24]	€17.42	
Neurologist visit	Costs per neurologist visit (specialist hospital, indexed to 2020) [24]	€113.20	
Triptan costs	Weighted average costs* per triptan [25, 26]	€0.39	
Travel expenses	Travel costs calculated at €0.19 per km with €3.00 parking costs and assuming 50% of patients use public transport, with the other 50% using their own transport. Distances used were as follows: GP visit, 2.2 km (round trip); neurologist visit, 14 km (round trip) [24]	GP visit: €3.70 Neurologist visit: €6.12	
Resource use – indirect costs			
Mean hourly wage	Average hourly wages were calculated based on Statistics Netherlands data, accounting for the sex balance and employment rates in each age group [27, 28]	Age	Wage
		> 45–50 years	€22.08
		> 50–55 years	€21.70
		> 55–60 years	€19.68
		> 60–65 years	€14.57
		> 65–70 years	€3.67
		> 70–75 years	€1.34
		> 75 years	€0.11

Weighted average cost was calculated by multiplying units dispensed of each triptan in the Netherlands in a year by the corresponding unit price, summing across triptans, and then dividing by total units of triptans dispensed. GP, general practitioner

### Main model base-case assumptions

The main model assumptions applied in the base case analysis are summarized in Table 2 [4, 14, 17, 20, 22, 24, 27–32]. Standard Dutch discount rates for costs and quality-adjusted life-years (QALYs) were applied [20]. Discontinuation was modeled in two ways based on FOCUS data; a rate of 0.51% per cycle was applied over the initial 12-week period, and then 1% of patients discontinued treatment due to adverse events after each 6-monthly monitoring break [14]. Treatment discontinuation due to age was included to reflect the decline in active migraine above the age of 55 years [4]. This was modeled as a 0.45% discontinuation per cycle between the ages of 55 and 75 years to equalize MMD between arms in these patients. MMD after therapy discontinuation were assumed to return to BSC MMD levels. No waning in treatment effect was modeled in the base case.

**Table 2:** Analysis Assumptions

Variable	Value
**Base case**	
Time horizon	Lifetime (to 100 years of age), to ensure all costs and benefits are captured
Intervention	Fremanezumab
Comparator	BSC (acute migraine treatment only)
Perspective	Societal
Population	DPG: patients with CM who have had an inadequate response to topiramate or valproate and onabotulinumtoxinA FOCUS CM population: patients with CM who have had an inadequate response to 2–4 classes of migraine preventive therapy
Discount rates	Costs, 4%; QALYs, 1.5%, as per ZIN guidance [20]
Model cycle length	4 weeks, to match FOCUS trial assessment periods [14]
Patient baseline characteristics	Mean age, 47 years; proportion female, 82.3%
MMD distribution	Beta binomial distributions [38]
MMD reduction	Treatment effect based on difference vs placebo from FOCUS data [14]
Response rate	DPG: BSC, 13%; fremanezumab, 41% [17] FOCUS CM population: BSC, 16%; fremanezumab, 51% [14]
Long-term efficacy	Data from the HALO trial extension showed efficacy is maintained at similar levels for up to 15 months (currently available data) [22]; the model therefore assumes that the modeled migraine days at Week 12 are maintained for the rest of the time horizon
Utilities	MSQ from FOCUS data mapped to EQ-5D-3L [14]
Resource use	National Health and Wellness Survey [29, 30]
Resource costs	*Manual for Cost Research* [24]
Productivity losses	Total number of missed workdays (due to absenteeism and presenteeism, assuming 50% productivity losses on presenteeism days) [31]
Productivity costs	Hourly wage per sex and age from Netherlands statistics [27, 28]
Adverse events	Adverse events associated with fremanezumab were infrequent, were usually not severe, and occurred at rates that were comparable to those seen with placebo. It was assumed that no resource use, and therefore no costs or disutility, would be associated with adverse events
Negative stopping rule	Patients who do not respond to treatment (a ≥ 30% reduction) stop after 12-week assessment
Discontinuation during 12-week trial period	A rate of 0.51% per cycle was applied over the initial 12-week period based on FOCUS trial data [14]
Long-term discontinuation	1% discontinuation due to adverse events after each 6-monthly monitoring based on FOCUS trial extension data
Treatment discontinuation due to age	0.45% discontinuation per cycle between the ages of 55–75 years. After the age of 55 years, the percentage of active patients with migraine decreases sharply, from around 15% to around 5% at the age of 75 years [4]. This reduction was modeled as a continuous and gradual reduction
MMD after therapy discontinuation	After negative stopping rule: return to BSC MMD After long-term monitoring discontinuation: return to BSC MMD Treatment discontinuation due to age: return to BSC MMD (i.e., equal MMD between arms are assumed for these patients)
Waning	No waning in treatment effect occurs; available evidence shows maintenance of treatment effect for up to 15 months (with currently available data) [22]
Mortality	No migraine-specific mortality; baseline mortality based on age and sex only
**Scenario analyses**	
Utilities using direct EQ-5D-5L data	The directly collected EQ-5D-5L data from the FOCUS trial [14] were utilized as an alternative set of utilities; however, these data did not appear to capture the full burden of migraine, and hence the MSQ data were preferred in the base case
Long-term discontinuation scenarios	As the rate of long-term discontinuation is based on limited current data, the impact of this input was investigated by including scenarios with no long-term discontinuation and a higher rate of long-term discontinuation (2% or 4%, respectively, at each assessment)
Discontinuation due to age scenarios	To assess the impact of this input, additional scenarios without discontinuation due to age and an increased rate of discontinuation (1% per cycle)
Hospitalization and emergency department visit costs included	Costs for hospitalization and emergency department visits included, with resource use data from the National Health and Wellness Survey [29, 30] and costs from the Dutch guide *Kostenhandleiding* [24]
Inclusion of consideration of Netherlands sickness law	Work loss in high-MMD states would be covered under Netherlands sickness law, and so workday loss reduced by 50% for > 16 MMD
Inclusion of informal care costs	Patients with migraine may sometimes need help to complete household tasks when suffering a migraine attack. For this scenario, it was estimated 50% of patients require informal care [32]; time requirement were estimated as half of a working day (4 h) of informal care on a migraine day
Healthcare perspective	Analysis conducted from a healthcare system perspective using direct costs only
Time horizon scenarios	Scenarios with time horizons of 2, 5, and 10 years were included to illustrate outcomes over time frames shorter than lifetime
Equal utilities between on and off treatment	In the base case, a treatment effect is applied to the utilities based on the significance of this effect in statistical analyses; this scenario investigates the impact of removing this effect and equalizing utilities between on- and off-treatment patients
Treatment waning	The treatment effect of fremanezumab reduced linearly over a period of 10 years (after the initial 12-week assessment period), so that, at the end of the period, average MMD are equal between fremanezumab and BSC

BSC, best supportive care; CM, chronic migraine; DPG, Dutch patient group; MMD, monthly migraine days; MSQ, Migraine-Specific Quality of Life Questionnaire; QALY, quality-adjusted life-year; ZIN, Zorginstituut Nederland

### Model outputs

The model produces outputs of costs and QALYs for each intervention. Incremental differences in costs and QALYs and the associated incremental cost-effectiveness ratios (ICERs) are presented as primary model results.

### Sensitivity analyses

A one-way sensitivity analysis (OWSA) and a probabilistic sensitivity analysis (PSA) were conducted. For the OWSA, all main inputs were varied by ± 20% to illustrate the model’s sensitivity to individual inputs. The PSA utilized random values for inputs based on normal, beta, or gamma distributions (as appropriate) and with an assumption of a 20% variance. The PSA was performed by running 10,000 Monte Carlo simulations and using a €50,000/QALY willingness-to-pay (WTP) threshold; this threshold is based on the disease burden calculated using the Institute for Medical Technology Assessment Disease Burden Calculator. For this model, the proportional shortfall was 0.53, which corresponds to a WTP threshold of €50,000/QALY. Full details on the OWSA and PSA inputs are included in Table S4. Scenario analyses were included to investigate key individual inputs, and these, including the associated rationales, are summarized in Table 2.

## Results

In the base case (Table 3), fremanezumab is cost-saving compared with BSC (saving of €2514). In addition, HRQoL benefits for fremanezumab treatment were clearly demonstrated, with decreased migraine days leading to an increase in QALYs of 1.45 compared with BSC (12.80 vs 11.35). This made fremanezumab the dominant (less cost and higher QALY gains) treatment option. In the wider FOCUS CM population (Table 3), fremanezumab led to a larger increase in QALYs of 1.82 that was associated with additional costs of €4624, leading to an ICER of €2547/QALY.

**Table 3:** Base-case Model Results

	BSC	Fremanezumab
**DPG**		
**Total costs (€)**	€161,554	€159,040
*Preventive treatment costs (€)*	*€0*	*€24,868*
*Monitoring costs (€)*	*€0*	*€1066*
*Resource use costs (€)*	*€8108*	*€7502*
*Productivity costs (€)*	*€153,447*	*€125,603*
**Incremental costs (€)**	–	**–€2514**
**Total QALYs**	11.35	12.80
**Incremental QALYs**	–	**1.45**
**ICER vs BSC (€/QALY)**	–	**Dominant**
**FOCUS CM population**		
**Total costs (€)**	€127,743	€132,368
*Preventive treatment costs (€)*	*€0*	*€30,500*
*Monitoring costs (€)*	*€0*	*€1312*
*Resource use costs (€)*	*€7802*	*€6782*
*Productivity costs (€)*	*€119,942*	*€93,773*
**Incremental costs (€)**	–	**€4624**
**Total QALYs**	12.55	14.37
**Incremental QALYs**	–	**1.82**
**ICER vs BSC (€/QALY)**	–	**€2547**

BSC, best supportive care; CM, chronic migraine; DPG, Dutch patient group; ICER, incremental cost-effectiveness ratio; QAL, quality-adjusted life-year

**Fig. 2:**
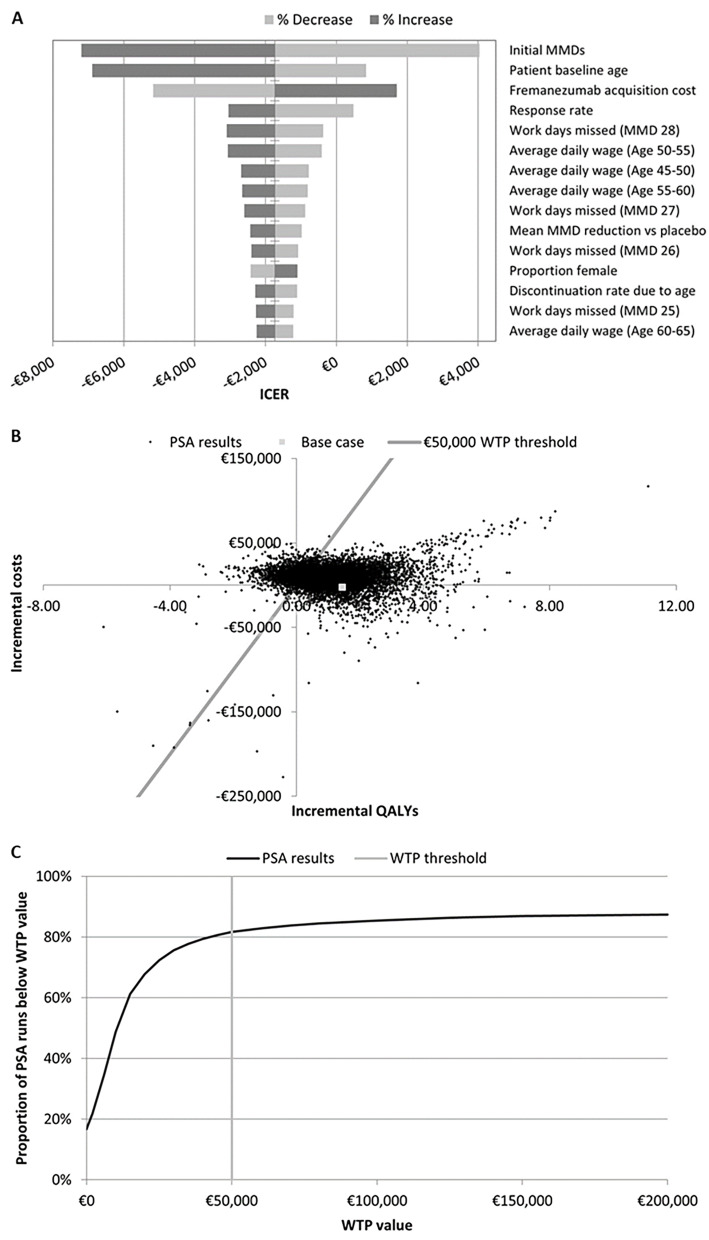
Sensitivity analysis results. Part **A** shows a tornado plot of deterministic sensitivity analysis results. Parts **B** and **C**, respectively, show a scatter plot of and WTP curve for PSA results. ICER, incremental cost-effectiveness ratio; MMD, monthly migraine days; PSA, probabilistic sensitivity analysis; QALY, quality-adjusted life-year; WTP, willingness-to-pay

The OWSA (Fig. 2) showed initial MMD (–€7201/QALY to €4035/QALY), starting age (–€6893/QALY to €842/QALY), fremanezumab cost (–€5173/QALY to €1699/QALY), and response rate (–€3042/QALY to €477/QALY) were the inputs with the greatest impact on model outcomes. In all cases, fremanezumab remained highly cost effective compared with BSC, and results were consistent in the FOCUS CM population (Figure S2). The PSA (Fig. 2) showed a relatively large spread in results, but over 10,000 iterations, 76.1% were below the €50,000/QALY WTP threshold in the DPG. Further investigations revealed the time horizon was a major contributor to the large spread (additional results in Figure S2).

A number of key scenarios were investigated, and fremanezumab remained dominant or cost-effective in all of them (Table 4). Of particular note is when productivity losses are excluded, the ICER rises considerably to €17,498/QALY (€16,959/QALY in the FOCUS CM population); but even then, the highest ICER produced in these scenarios would be considered a cost-effective result. Also noteworthy is that, when informal care costs are included, the potential cost-savings associated with use of fremanezumab are substantially larger, with fremanezumab becoming a dominant strategy in the FOCUS CM population. Fremanezumab also remains dominant or cost-effective when direct EQ-5D-5L data are used (€5316/QALY gained in the FOCUS CM population) and under shorter time horizons in the FOCUS CM population (€13,268, €7389, and €4620/QALY gained over 2-year, 5-year, and 10-year time horizons, respectively).

**Table 4:** Scenario Analyses

	Incremental costs (€)	Incremental QALYs	ICER vs BSC (€/QALY)
**Primary study population**			
*Base case*	*–€2514*	*1.45*	*Dominant*
Utilities using direct EQ-5D-5L data	–€2514	0.80	Dominant
No long-term discontinuation	€6300	1.55	€4058
Long-term discontinuation rate set to 2% at each assessment	–€5374	1.37	Dominant
Long-term discontinuation rate set to 4% at each assessment	–€9175	1.27	Dominant
No discontinuation due to age	€4190	1.62	€2579
Discontinuation due to age raised to 1%	–€5767	1.36	Dominant
Costs for hospitalization and emergency department visits included	–€3265	1.45	Dominant
Inclusion of consideration of Netherlands sickness law	€16,750	1.45	€11,571
Inclusion of carer costs/informal care	–€25,919	1.45	Dominant
Healthcare perspective	€25,329	1.45	€17,498
Time horizon, 2 years	€828	0.14	€5760
Time horizon, 5 years	–€300	0.35	Dominant
Time horizon, 10 years	–€2042	0.65	Dominant
Equal utilities between on and off treatment	–€2514	1.26	Dominant
Treatment waning	€922	1.24	€744
**FOCUS CM patients**			
*Base case*	*€4624*	*1.82*	*€2547*
Utilities using direct EQ-5D-5L data	€4624	0.87	€5316
No long-term discontinuation	€16,502	1.88	€8770
Long-term discontinuation rate set to 2% at each assessment	€323	1.77	€183
Long-term discontinuation rate set to 4% at each assessment	–€5544	1.70	Dominant
No discontinuation due to age	€13,695	1.93	€7107
Discontinuation due to age raised to 1%	€24	1.76	€13
Costs for hospitalization and emergency department visits included	€3409	1.82	€1878
Inclusion of consideration of Netherlands sickness law	€22,795	1.82	€12,555
Inclusion of carer costs/informal care	–€24,618	1.82	Dominant
Healthcare perspective	€30,793	1.82	€16,959
Time horizon, 2 years	€2147	0.16	€13,268
Time horizon, 5 years	€2899	0.39	€7389
Time horizon, 10 years	€3415	0.74	€4620
Equal utilities between on and off treatment	€4624	1.57	€2936
Treatment waning	€5311	1.77	€3004

BSC, best supportive care; CM, chronic migraine; ICER, incremental cost-effectiveness ratio; QALY, quality-adjusted life-year

## Discussion

This novel analysis demonstrates fremanezumab is cost saving in the Dutch CM subgroup of patients with inadequate response to prior migraine preventive treatments. This represents the first fully published cost-effectiveness analysis for fremanezumab. This analysis adds to growing cost-effectiveness evidence for CGRP pathway mAbs within a European context. It is conducted fully in line with guidance of the Dutch Health Technology Agency, which has led to an analysis using a more conservative set of assumptions compared with many other economic analyses in migraine [18, 33–36]. This cost-effectiveness analysis is the first to utilize data from the FOCUS trial, which was specifically focused on patients with an inadequate response to 2–4 classes of previous migraine preventive treatments.

The analysis in the FOCUS CM population showed greater QALY benefits could be achieved in this wider population at additional cost but with fremanezumab remaining cost effective. This suggests the currently reimbursed population within the Netherlands is well targeted to provide a high level of cost effectiveness. Treatment of a population equivalent to the full FOCUS CM population could lead to greater patient benefits whilst maintaining cost effectiveness.

These results also have applicability across European countries with comparable healthcare systems. Of the most interest is the FOCUS CM population, which demonstrates more patient benefits. In many areas, choices made during model construction have been conservative to match Dutch requirements (e.g., not including hospital admission or emergency room costs). Whilst exact results will vary depending on local assumptions and cost sources, results herein show fremanezumab is likely to be cost effective for the treatment of CM across similar European countries. Within the literature, a number of economic analyses of migraine preventive therapies have been conducted within Europe [18, 33–36]. These analyses have primarily focused on onabotulinumtoxinA [33–36], with a single report investigating an alternative CGRP pathway mAb (erenumab) in Sweden [18]. These analyses have utilized a variety of country-specific assumptions and populations, making direct comparison challenging. However, results were broadly consistent between studies. When indirect costs were considered, preventive treatments were generally dominant (more effective and less costly) compared with BSC [18, 36]. When indirect costs were excluded, ICER values were generally in the range of €15,000/QALY to €25,000/QALY versus BSC [18, 33, 35, 36]. The study with the greatest relevance is that considering erenumab, which again had broadly comparable results but a positive stopping rule (a stopping rule for responder patients), was used for modeling erenumab [18]. Such rules have generally seldom been acceptable to European health technology agencies due to a current lack of supporting evidence. Our model did not utilize a positive stopping rule, exemplifying its conservative nature.

The sensitivity analyses showed the model was robust to changes in major inputs, with no OWSA or scenario analysis producing an ICER over the €50,000/QALY WTP threshold. The PSA showed a relatively high variance, which was mainly attributable to the lifetime horizon. The use of inputs for utility and workday impacts of individual MMD was implemented based on initial guidance by ZIN and represents an approach likely to overestimate variance. There is a strong correlation between these closely related states (which differ by a single migraine day); thus, individually varying these inputs could potentially lead to an overestimation of the total variance. Considering these factors, ours is a robust model focused on the key population of interest.

Migraine is a complex disease for economic modeling, but this model has been built to match clinical aspects of this disease as accurately as possible. Migraine is a long-term condition that is not degenerative, so an individual patient can potentially improve or worsen over the long-term. Whilst this natural history variation in migraine is generally known, there is a lack of studies of sufficient detail to fully include this within economic models. However, an age discontinuation was added to this model to reflect the decline in migraine prevalence above the age of 55 years [4]. This was applied to fremanezumab-treated patients to ensure patients who no longer have active migraine were not treated and costs and QALYs were equal between arms. This approach did not, however, correct absolute QALY values as patients were still modeled to experience MMD. This means this factor has not been fully included but has been considered in more detail than in other migraine models [18, 33–36]. Another aspect of note is that this model allows patients to experience all possible MMD values. This allows the model a high degree of fidelity to account for changes in the distribution of patients across MMD states whilst also fully reflecting patient experience. Another novel addition to reflect the clinical situation of the Netherlands was long-term discontinuation due to adverse events during 6-monthly monitoring visits. The response assessment was modeled to be at 12 weeks, in line with clinical trial assessment. However, the reimbursement criteria in this particular case are provided with an additional practice-based criteria set for neurologists to allow continuation of the treatment in individual patient cases when a reduction of 30% has been reached in at least half of the months during a 6-month period.

A limitation of this model, in common with other economic models for migraine, is that the model is focused on MMD as the driver of severity. Whilst MMD are a key driver of migraine severity, this focus does not necessarily capture the full impacts of migraine. In particular, this approach means the severity of attacks, burden on patients between attacks, and psychological impacts are not fully captured. This makes it likely that the true burden of migraine is higher than what we have modeled in this study, with the possibility that the benefits of fremanezumab may also be greater if treatment impacts these factors.

A further limitation is the long-term uncertainty associated with currently available data for this relatively novel therapy. It must be noted that although no adverse safety signals have been observed in current data, the potential for adverse events and complications following extended long-term treatment are not yet known. However, analyses with shorter time horizons highlight that the cost effectiveness of fremanezumab can be shown at time scales where there is reduced long-term uncertainty. The primary patient population results rely on data from a post hoc subgroup analysis of the FOCUS trial [17]. This leads to some uncertainty due to the reduced group size available (138 patients); also 40–60% of patients had inadequate response to four prior migraine preventive medication classes [17], meaning additional failures beyond Dutch requirements (mostly beta-blockers and/or tricyclic antidepressants). To address these uncertainties, an additional supportive analysis was conducted within the full FOCUS CM population (509 patients). This produced results consistent with the DPG results and supports the results. This model was utilized as the basis for the reimbursement application for the Netherlands and thus has been thoroughly reviewed by ZIN. The results of these analyses were found to be cost-effective and allowed reimbursement to be approved for the primary population of CM patients who have failed on prior preventive therapy [37].

Whenever data from multinational clinical trials are used in country-specific models, the question often arises as to how representative the trial data are of the local population. This can be a particular concern when the indication studied in the clinical trial differs from how a product may be used post market authorization. We employed data from the multinational FOCUS study in our model, which included sites in the Netherlands. As part of the Dutch health technology assessment process an external clinical advisory panel was consulted and asked whether, in its opinion, the FOCUS population was comparable to the treatment-eligible Dutch population [38]. The panel considered the FOCUS population to be comparable to the population considered for treatment in the Netherlands. We further note that the subgroup of CM subjects in FOCUS who had failed topiramate or valproate plus onabotulinumtoxinA are the patients who will be treated in the Netherlands.

Finally, we note that we did not account for possible intervariable effects in the PSA. To the extent such effects are manifest in the data used to populate the model variables included in the PSA, our findings may be affected. However, we note that our PSA findings trended strongly toward cost-effectiveness at the €50,000 per QALY gained threshold, with more than three-quarters of iterations falling below this threshold, and thus intervariable effects may not have material impact on these findings.

## Conclusions

Within the Dutch population of CM patients who have failed on prior preventive therapy fremanezumab is a cost-saving treatment that leads to increased QALYs compared with BSC. Fremanezumab was cost effective versus BSC in a wider CM group of ≥ 2 failures. Together, this demonstrates fremanezumab is a cost-effective treatment for migraine prevention in patients with CM.

### Electronic supplementary material

Below is the link to the electronic supplementary material.

Supplementary Material 1

## Data Availability

The data that support the findings of this study are available from the corresponding author upon reasonable request.

## Abbreviations

BIC: Bayesian Information Criterion
BSC: best supportive care
CGRP: calcitonin gene-related peptide
CHEERS: Consolidated Health Economic Evaluation Reporting Standards
CM: chronic migraine
DPG: Dutch patient group
EM: episodic migraine
GP: general practitioner
HRQoL: health-related quality of life
ICER: incremental cost-effectiveness ratio
mAb: monoclonal antibody
MMD: monthly migraine days
MSQ: Migraine-Specific Quality of Life Questionnaire
OWSA: one-way sensitivity analysis
PSA: probabilistic sensitivity analysis
QALY: quality-adjusted life years
WTP: willingness-to-pay
ZIN: Zorginstituut Nederland
