# Supplementary material for: A Dutch cost-effectiveness analysis of fremanezumab versus best supportive care in patients with chronic migraine and inadequate response to prior preventive therapy
Source: BMC Neurol. 2024 Jun 24;24:214. doi: 10.1186/s12883-024-03697-x (PMC11194870; doi:10.1186/s12883-024-03697-x)
Supplement: Supplementary file 1 — Supplementary Material 1 [file 12883_2024_3697_MOESM1_ESM.docx]

**Supplementary Materials**

**Table S1** Sigma Parameters and Goodness of Fit for CM Responders

| **Distribution** | **Placebo** | **Fremanezumab** |  | **AIC** | **BIC** |
| --- | --- | --- | --- | --- | --- |
| Beta Binomial  (Intra class correlation [ρ]) | 0.038 | 0.079 |  | 3258 | 3343 |
| Negative Binomial  (Dispersion [φ]) | 0.052 | 0.159 |  | 3297 | 3332 |

A responder was a patient with CM with a ≥30% reduction in MMD from baseline during the 12 weeks after therapy initiation. AIC, Akaike information criterion; BIC, Bayesian information criterion; CM, chronic migraine; MMD, monthly migraine days.

From ‘Farmaco-economisch rapport voor fremanezumab (Ajovy®) bij de profylaxe van chronische migraine bij volwassenen nadat zij op topiramaat of valproaat en botulinetoxine A hebben gefaald’. Zorginstituut Nederlands (2021).

**Table S2.** Sigma Parameters and Goodness of Fit for CM Non-Responders

| **Distribution** | **Placebo** | **Fremanezumab** | **AIC** | **BIC** |
| --- | --- | --- | --- | --- |
| Beta Binomial  (Intra class correlation [ρ]) | 0.096 | 0.092 | 4821 | 4934 |
| Negative Binomial  (Dispersion [φ]) | 0.000 | 0.000 | 5119 | 5159 |

AIC, Akaike information criterion; BIC, Bayesian information criterion; CM, chronic migraine.

From ‘Farmaco-economisch rapport voor fremanezumab (Ajovy®) bij de profylaxe van chronische migraine bij volwassenen nadat zij op topiramaat of valproaat en botulinetoxine A hebben gefaald’. Zorginstituut Nederlands (2021).

**Table S3** Utility Value Inputs

| **MMD** | **MSQ-derived utility values** | | **EQ-5D-5L utility values** | |
| --- | --- | --- | --- | --- |
|  | **Off treatment** | **On treatment** | **Off treatment** | **On treatment** |
| 0 | 0.758 | 0.795 | 0.951 | 0.960 |
| 1 | 0.745 | 0.782 | 0.947 | 0.956 |
| 2 | 0.733 | 0.770 | 0.943 | 0.953 |
| 3 | 0.719 | 0.756 | 0.939 | 0.949 |
| 4 | 0.706 | 0.743 | 0.934 | 0.944 |
| 5 | 0.692 | 0.728 | 0.929 | 0.940 |
| 6 | 0.677 | 0.713 | 0.924 | 0.935 |
| 7 | 0.662 | 0.698 | 0.919 | 0.929 |
| 8 | 0.647 | 0.682 | 0.913 | 0.923 |
| 9 | 0.631 | 0.666 | 0.907 | 0.917 |
| 10 | 0.616 | 0.649 | 0.900 | 0.910 |
| 11 | 0.599 | 0.632 | 0.893 | 0.903 |
| 12 | 0.583 | 0.614 | 0.886 | 0.895 |
| 13 | 0.566 | 0.596 | 0.878 | 0.887 |
| 14 | 0.550 | 0.578 | 0.870 | 0.878 |
| 15 | 0.533 | 0.560 | 0.861 | 0.868 |
| 16 | 0.516 | 0.541 | 0.851 | 0.858 |
| 17 | 0.498 | 0.522 | 0.841 | 0.847 |
| 18 | 0.481 | 0.503 | 0.831 | 0.836 |
| 19 | 0.464 | 0.484 | 0.820 | 0.824 |
| 20 | 0.447 | 0.465 | 0.809 | 0.811 |
| 21 | 0.430 | 0.446 | 0.797 | 0.797 |
| 22 | 0.413 | 0.427 | 0.784 | 0.783 |
| 23 | 0.396 | 0.408 | 0.771 | 0.767 |
| 24 | 0.379 | 0.390 | 0.757 | 0.751 |
| 25 | 0.362 | 0.371 | 0.743 | 0.735 |
| 26 | 0.346 | 0.353 | 0.728 | 0.718 |
| 27 | 0.330 | 0.335 | 0.712 | 0.700 |
| 28 | 0.314 | 0.318 | 0.696 | 0.681 |

MMD, monthly migraine days; MSQ, Migraine-Specific Quality of Life Questionnaire.

**Table S4** Sensitivity Analysis Inputs

| **Parameter** | **Inclusion in OWSA** | **OWSA variation** | **Inclusion in PSA** | **Distribution type used in PSA** |
| --- | --- | --- | --- | --- |
| Patient baseline age | Yes | ±20% | Yes | Normal |
| Proportion female | Yes | ±20% | Yes | Normal |
| Fremanezumab acquisition cost | Yes | ±20% | Yes | Gamma |
| Monitoring costs | Yes | ±20% | Yes | Gamma |
| Discontinuation rate during  12-week trial period | Yes | ±20% | Yes | Normal |
| Discount rates | Yes | 0 to –100% | No | – |
| Analysis time horizon (number of model cycles) | Yes | ±20% | No | – |
| Initial MMD | Yes | ±20% | Yes | Normal |
| GP visit cost | Yes | ±20% | Yes | Gamma |
| Triptan cost | Yes | ±20% | Yes | Gamma |
| Neurologist visit cost | Yes | ±20% | Yes | Gamma |
| Mean MMD reduction vs placebo | Yes | ±20% | Yes | Normal |
| Response rate | Yes | ±20% | Yes | Normal |
| Long-term discontinuation rate | Yes | ±20% | Yes | Normal |
| Discontinuation rate due to age | Yes | ±20% | Yes | Normal |
| Resource use multiplier | Yes | ±20% | Yes | Normal |
| On-treatment utility (MMD 0) | Yes | ±20% | Yes | Beta |
| On-treatment utility (MMD 1) | Yes | ±20% | Yes | Beta |
| On-treatment utility (MMD 2) | Yes | ±20% | Yes | Beta |
| On-treatment utility (MMD 3) | Yes | ±20% | Yes | Beta |
| On-treatment utility (MMD 4) | Yes | ±20% | Yes | Beta |
| On-treatment utility (MMD 5) | Yes | ±20% | Yes | Beta |
| On-treatment utility (MMD 6) | Yes | ±20% | Yes | Beta |
| On-treatment utility (MMD 7) | Yes | ±20% | Yes | Beta |
| On-treatment utility (MMD 8) | Yes | ±20% | Yes | Beta |
| On-treatment utility (MMD 9) | Yes | ±20% | Yes | Beta |
| On-treatment utility (MMD 10) | Yes | ±20% | Yes | Beta |
| On-treatment utility (MMD 11) | Yes | ±20% | Yes | Beta |
| On-treatment utility (MMD 12) | Yes | ±20% | Yes | Beta |
| On-treatment utility (MMD 13) | Yes | ±20% | Yes | Beta |
| On-treatment utility (MMD 14) | Yes | ±20% | Yes | Beta |
| On-treatment utility (MMD 15) | Yes | ±20% | Yes | Beta |
| On-treatment utility (MMD 16) | Yes | ±20% | Yes | Beta |
| On-treatment utility (MMD 17) | Yes | ±20% | Yes | Beta |
| On-treatment utility (MMD 18) | Yes | ±20% | Yes | Beta |
| On-treatment utility (MMD 19) | Yes | ±20% | Yes | Beta |
| On-treatment utility (MMD 20) | Yes | ±20% | Yes | Beta |
| On-treatment utility (MMD 21) | Yes | ±20% | Yes | Beta |
| On-treatment utility (MMD 22) | Yes | ±20% | Yes | Beta |
| On-treatment utility (MMD 23) | Yes | ±20% | Yes | Beta |
| On-treatment utility (MMD 24) | Yes | ±20% | Yes | Beta |
| On-treatment utility (MMD 25) | Yes | ±20% | Yes | Beta |
| On-treatment utility (MMD 26) | Yes | ±20% | Yes | Beta |
| On-treatment utility (MMD 27) | Yes | ±20% | Yes | Beta |
| On-treatment utility (MMD 28) | Yes | ±20% | Yes | Beta |
| Off-treatment utility (MMD 0) | Yes | ±20% | Yes | Beta |
| Off-treatment utility (MMD 1) | Yes | ±20% | Yes | Beta |
| Off-treatment utility (MMD 2) | Yes | ±20% | Yes | Beta |
| Off-treatment utility (MMD 3) | Yes | ±20% | Yes | Beta |
| Off-treatment utility (MMD 4) | Yes | ±20% | Yes | Beta |
| Off-treatment utility (MMD 5) | Yes | ±20% | Yes | Beta |
| Off-treatment utility (MMD 6) | Yes | ±20% | Yes | Beta |
| Off-treatment utility (MMD 7) | Yes | ±20% | Yes | Beta |
| Off-treatment utility (MMD 8) | Yes | ±20% | Yes | Beta |
| Off-treatment utility (MMD 9) | Yes | ±20% | Yes | Beta |
| Off-treatment utility (MMD 10) | Yes | ±20% | Yes | Beta |
| Off-treatment utility (MMD 11) | Yes | ±20% | Yes | Beta |
| Off-treatment utility (MMD 12) | Yes | ±20% | Yes | Beta |
| Off-treatment utility (MMD 13) | Yes | ±20% | Yes | Beta |
| Off-treatment utility (MMD 14) | Yes | ±20% | Yes | Beta |
| Off-treatment utility (MMD 15) | Yes | ±20% | Yes | Beta |
| Off-treatment utility (MMD 16) | Yes | ±20% | Yes | Beta |
| Off-treatment utility (MMD 17) | Yes | ±20% | Yes | Beta |
| Off-treatment utility (MMD 18) | Yes | ±20% | Yes | Beta |
| Off-treatment utility (MMD 19) | Yes | ±20% | Yes | Beta |
| Off-treatment utility (MMD 20) | Yes | ±20% | Yes | Beta |
| Off-treatment utility (MMD 21) | Yes | ±20% | Yes | Beta |
| Off-treatment utility (MMD 22) | Yes | ±20% | Yes | Beta |
| Off-treatment utility (MMD 23) | Yes | ±20% | Yes | Beta |
| Off-treatment utility (MMD 24) | Yes | ±20% | Yes | Beta |
| Off-treatment utility (MMD 25) | Yes | ±20% | Yes | Beta |
| Off-treatment utility (MMD 26) | Yes | ±20% | Yes | Beta |
| Off-treatment utility (MMD 27) | Yes | ±20% | Yes | Beta |
| Off-treatment utility (MMD 28) | Yes | ±20% | Yes | Beta |
| Workdays missed (MMD 0) | Yes | ±20% | Yes | Normal |
| Workdays missed (MMD 1) | Yes | ±20% | Yes | Normal |
| Workdays missed (MMD 2) | Yes | ±20% | Yes | Normal |
| Workdays missed (MMD 3) | Yes | ±20% | Yes | Normal |
| Workdays missed (MMD 4) | Yes | ±20% | Yes | Normal |
| Workdays missed (MMD 5) | Yes | ±20% | Yes | Normal |
| Workdays missed (MMD 6) | Yes | ±20% | Yes | Normal |
| Workdays missed (MMD 7) | Yes | ±20% | Yes | Normal |
| Workdays missed (MMD 8) | Yes | ±20% | Yes | Normal |
| Workdays missed (MMD 9) | Yes | ±20% | Yes | Normal |
| Workdays missed (MMD 10) | Yes | ±20% | Yes | Normal |
| Workdays missed (MMD 11) | Yes | ±20% | Yes | Normal |
| Workdays missed (MMD 12) | Yes | ±20% | Yes | Normal |
| Workdays missed (MMD 13) | Yes | ±20% | Yes | Normal |
| Workdays missed (MMD 14) | Yes | ±20% | Yes | Normal |
| Workdays missed (MMD 15) | Yes | ±20% | Yes | Normal |
| Workdays missed (MMD 16) | Yes | ±20% | Yes | Normal |
| Workdays missed (MMD 17) | Yes | ±20% | Yes | Normal |
| Workdays missed (MMD 18) | Yes | ±20% | Yes | Normal |
| Workdays missed (MMD 19) | Yes | ±20% | Yes | Normal |
| Workdays missed (MMD 20) | Yes | ±20% | Yes | Normal |
| Workdays missed (MMD 21) | Yes | ±20% | Yes | Normal |
| Workdays missed (MMD 22) | Yes | ±20% | Yes | Normal |
| Workdays missed (MMD 23) | Yes | ±20% | Yes | Normal |
| Workdays missed (MMD 24) | Yes | ±20% | Yes | Normal |
| Workdays missed (MMD 25) | Yes | ±20% | Yes | Normal |
| Workdays missed (MMD 26) | Yes | ±20% | Yes | Normal |
| Workdays missed (MMD 27) | Yes | ±20% | Yes | Normal |
| Workdays missed (MMD 28) | Yes | ±20% | Yes | Normal |
| Average daily wage  (Age >45–50 years) | Yes | ±20% | Yes | Gamma |
| Average daily wage  (Age >50–55 years) | Yes | ±20% | Yes | Gamma |
| Average daily wage  (Age >55–60 years) | Yes | ±20% | Yes | Gamma |
| Average daily wage  (Age >60–65 years) | Yes | ±20% | Yes | Gamma |
| Average daily wage  (Age >65–70 years) | Yes | ±20% | Yes | Gamma |
| Average daily wage  (Age >70–75 years) | Yes | ±20% | Yes | Gamma |
| Average daily wage  (Age >75 years) | Yes | ±20% | Yes | Gamma |

GP, general practitioner; MMD, monthly migraine days; OWSA, one-way sensitivity analysis; PSA, probabilistic sensitivity analysis.

**Fig. S1.** Distribution of 30% responders (**A**, **B**) and 30% non-responders (**C**, **D**) across the 29 health states for each treatment group at Week 0.


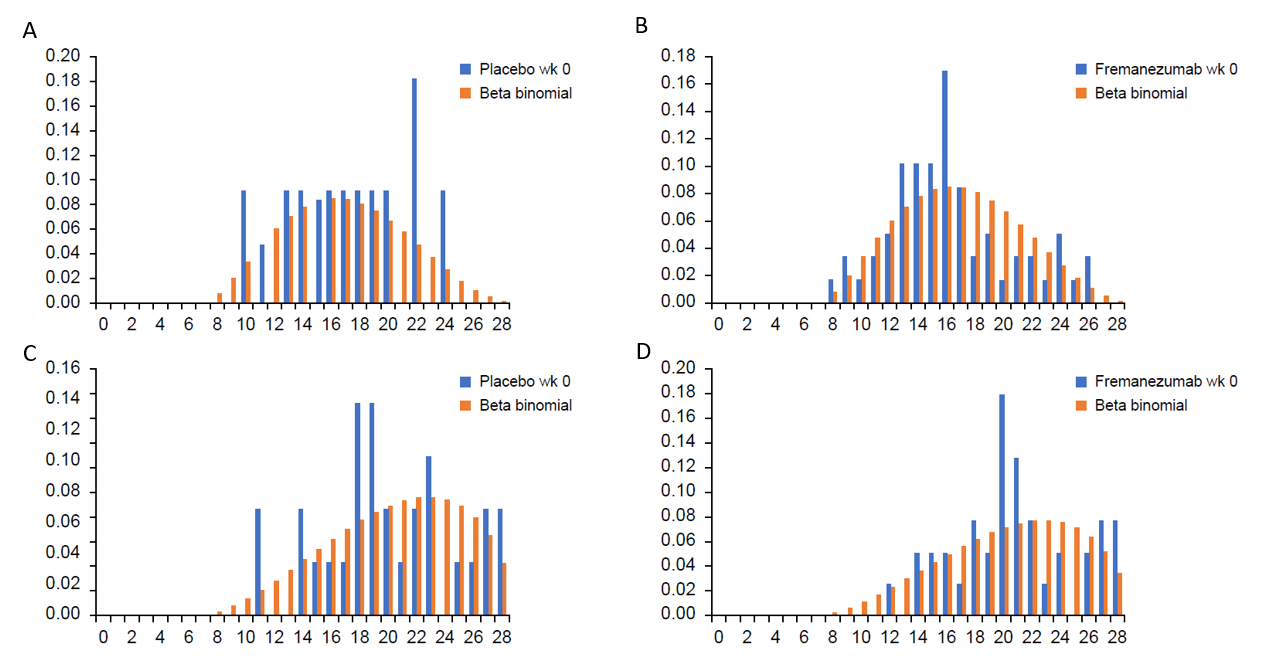


The observed study data at patient level (the blue bars) are displayed next to the fitted beta binomial model (the orange bars).

From ‘Farmaco-economisch rapport voor fremanezumab (Ajovy®) bij de profylaxe van chronische migraine bij volwassenen nadat zij op topiramaat of valproaat en botulinetoxine A hebben gefaald’. Zorginstituut Nederlands (2021).

**Fig. S2** Additional sensitivity analysis results. **A:** Scatter plot of PSA results for base case with 10-year time horizon. **B:** WTP curve for PSA results for base case with 10-year time horizon. **C:** Scatter plot of PSA results for full FOCUS CM population with lifetime horizon. **D:** WTP curve for PSA results for full FOCUS CM population. **E:** Tornado plot of deterministic sensitivity analysis results for full FOCUS CM population.


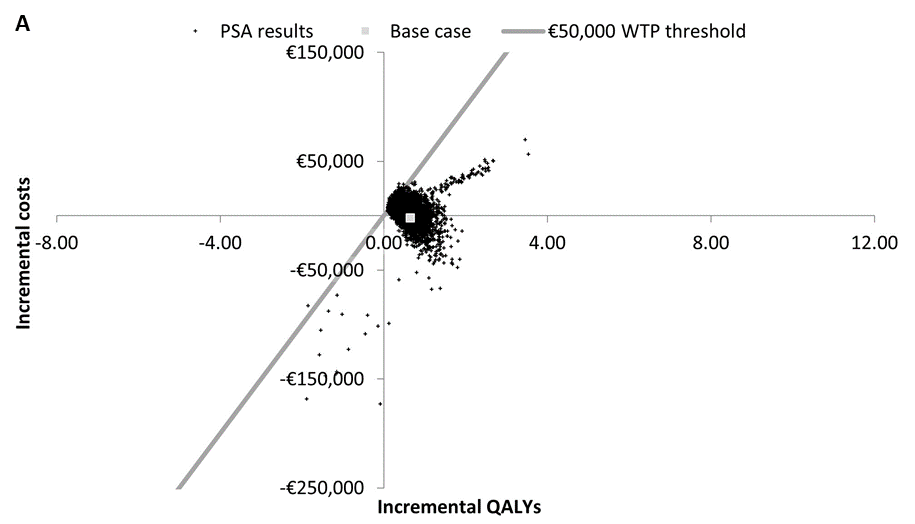


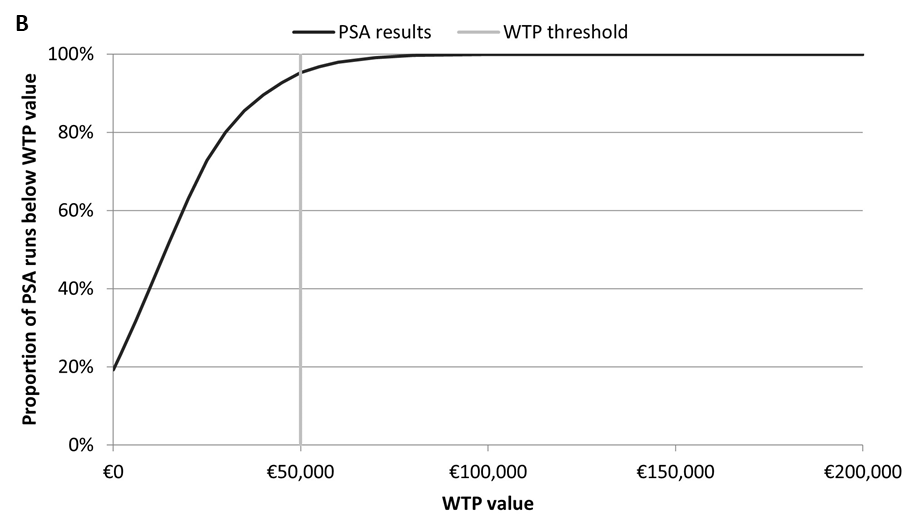


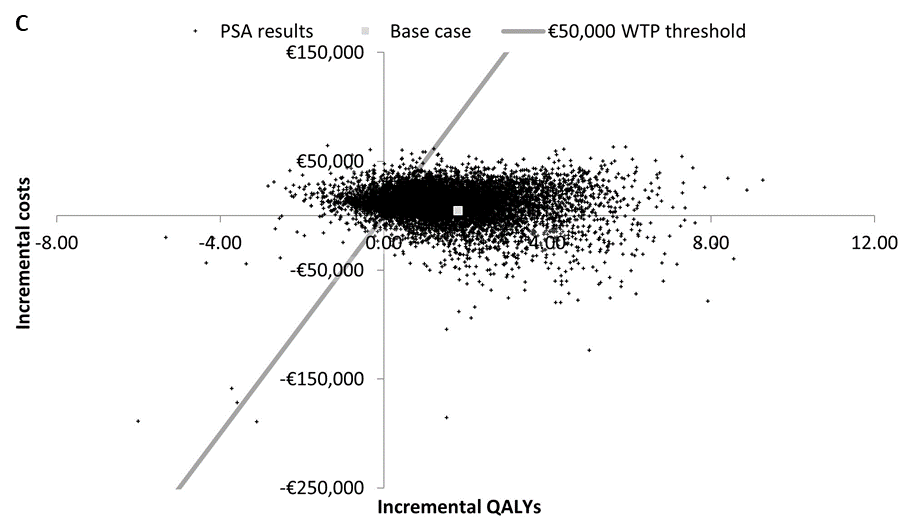


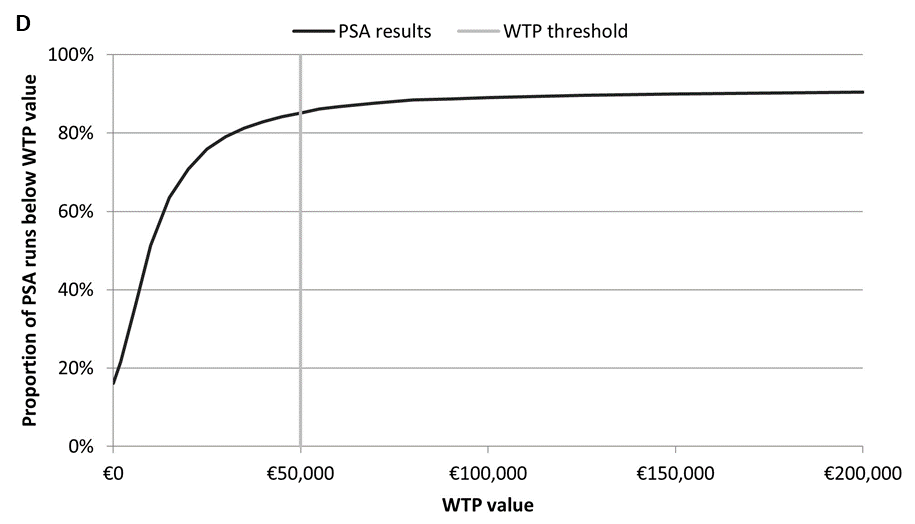


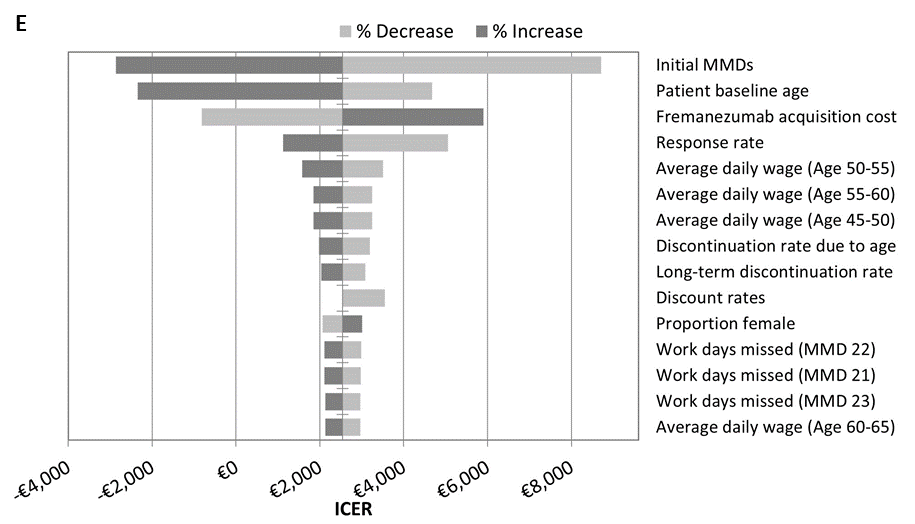


CM, chronic migraine; ICER, incremental cost-effectiveness ratio; MMD, monthly migraine days; PSA, probabilistic sensitivity analysis; QALY, quality-adjusted life-year; WTP, willingness-to-pay.
